# Basic processes as foundations of cognitive impairment in adult ADHD

**DOI:** 10.1007/s00702-019-02049-1

**Published:** 2019-07-18

**Authors:** Marah Butzbach, Anselm B. M. Fuermaier, Steffen Aschenbrenner, Matthias Weisbrod, Lara Tucha, Oliver Tucha

**Affiliations:** 1 Department of Clinical and Developmental Neuropsychology, Faculty of Behavioural and Social Sciences, University of Groningen, Grote Kruisstraat 2/1, 9712 TS Groningen, The Netherlands; 2 Department of Psychiatry and Psychotherapy, SRH Clinic Karlsbad-Langensteinbach, Karlsbad-Langensteinbach, Germany; 3 Department of General Psychiatry, Center of Psychosocial Medicine, University of Heidelberg, Heidelberg, Germany; 4 Department of Clinical Psychology and Neuropsychology, SRH Clinic Karlsbad-Langensteinbach, Karlsbad-Langensteinbach, Germany

**Keywords:** ADHD in adulthood, Cognitive impairment, Attention, Basic processes, Higher order functions, Processing speed

## Abstract

Attention deficit hyperactivity disorder (ADHD) in adulthood is associated with impairment of multiple aspects of cognition which adversely affect the individual’s everyday functioning. However, little is known about how these impairments are intertwined. This study explores whether impairments in basic processes (processing speed and distractibility) in adults with ADHD explain impairments in higher order functions, namely executive functions, memory, and complex attention. Furthermore, it is explored whether pharmacological treatment with methylphenidate (MPH) affects basic processes and higher order functions. A between-subjects design compared patients with ADHD without stimulant drug treatment (*N* = 55) and patients with ADHD treated with MPH (*N* = 31) with a healthy control group (*N* = 80). A neuropsychological test battery assessing basic processes and higher order functions was administered. Hierarchical logistic regression analyses were performed to evaluate the contribution of basic processes to impairments in higher order functions. Patients with ADHD not treated with MPH showed impairments in basic processes and higher order functions compared to controls. The impairments in basic processes explained 41–43% of impairments in executive functions, 27–29% in memory, and 56–74% in complex attention. In patients with ADHD treated with MPH, basic processes were not impaired and did not contribute significantly to impairments of higher order functions. Basic processes may constitute part of the foundation of cognitive impairments in adult ADHD. MPH may improve cognitive performance, presumably through improving basic processes. Applying this information could optimize neuropsychological assessments and inform treatment strategies by targeting basic processes.

## Introduction

Attention deficit/hyperactivity disorder (ADHD) is a neurodevelopmental disorder that in many cases persists into adulthood and that is associated with various impairments in everyday life, especially in the social and occupational realm (American Psychiatric Association 1994; Canu et al. 2008; Rösler et al. 2010). Adults with ADHD often have a lower quality of life, diminished educational achievement, lower employment rates, and are more likely to be involved in traffic accidents and encounters with the law (Agarwal et al. 2012; Rösler et al. 2010). Furthermore, ADHD in adulthood is characterized by deficits in cognitive functioning (Alderson et al. 2013; Boonstra et al. 2005; Fuermaier et al. 2014; Hervey et al. 2004; Rösler et al. 2010; Schoechlin and Engel 2005; Tucha et al. 2011). For example, attentional dysfunction has been consistently found in adults with ADHD (Hervey et al. 2004; Tucha et al. 2006, 2009) and whereas other core features of ADHD such as hyperactivity and impulsivity may diminish when children with ADHD reach adulthood, attentional dysfunction was reported to be stable over the lifespan (Hervey et al. 2004). Adults with ADHD are often reported to have deficits in executive functioning (Boonstra et al. 2005; Fuermaier et al. 2015), especially in working memory (Mostert et al. 2015; Wong and Stevens 2012), planning, and set shifting (Boonstra et al. 2005; Hervey et al. 2004). Impairments in executive functioning may play a central role in the cognition of adults with ADHD (Barkley and Murphy 1998); however, not all aspects of executive functioning seem to be equally impaired and not all patients show deficits in this domain (Bron et al. 2014; Hervey et al. 2004; Mostert et al. 2015). Finally, ADHD in adulthood has been linked to memory deficits across neuropsychological tests and across various aspects of memory, highlighting the pervasiveness of memory deficits in adults with ADHD (Fuermaier et al. 2013; Hervey et al. 2004).

ADHD appears to be cognitively heterogeneous (Mostert et al. 2015; Sonuga-Barke et al. 2010), which means that although patients with ADHD as a group show impairment in a given domain, there are large differences between patients, with about 11% of patients showing no cognitive impairment. The observed heterogeneity makes differentiating the role of specific cognitive functions difficult. To illustrate, an expert panel identified 26 neuropsychological functions as important in the assessment of adult ADHD (Fuermaier et al. 2019). One cannot assume, however, that every patient with ADHD will be impaired in all of these domains, as the type and extent of impairment varies considerably between patients (Mostert et al. 2015). Moreover, little is known about how the neuropsychological impairments associated with ADHD may interact to influence behavior. The variety of neuropsychological deficits found in adults with ADHD gave rise to the notion that impairments of attention could result in impaired performance of tests assessing other functions such as memory (Adams et al. 2011; Hervey et al. 2004; Mueller et al. 2017).

Basic processes may constitute a good starting point to investigate the notion how neuropsychological deficits arise and manifest in behavior. Impairments in processing speed and reaction time variability (RTV) are among the most robust findings and widely reported in the ADHD literature (Adams et al. 2011; Cross-Villasana et al. 2015; Kofler et al. 2013; Leth-Steensen et al. 2000; Mostert et al. 2015). Processing speed is commonly assessed by measuring participants’ reaction time (RT) to simple stimuli and patients with ADHD often show slower RTs than matched healthy control participants (Cross-Villasana et al. 2015; Kofler et al. 2013; Tucha et al. 2006). The slowing of RT in adult ADHD has been linked to abnormal neural activation at the stages of perception and response selection (Cross-Villasana et al. 2015). RTV is also of interest, as adults with ADHD have consistently shown more variability in their responding (Kofler et al. 2013) and increased RTV has even been labeled as a hallmark of ADHD (Mueller et al. 2017). RTV may arise through attentional lapses during task performance which result in a strongly skewed RT distribution (Gmehlin et al. 2016; Leth-Steensen et al. 2000; Mostert et al. 2015). Adams et al. (2011) directly tested the link between RTV and distractibility by tracking eye movements during an attentional task (stop-signal task), which reflects an individual’s ability to inhibit a reflexive response towards distracting stimuli. They found that for patients with ADHD, but not healthy controls, RTV is associated with distractibility and thus may represent a valid indicator of distractibility in adult ADHD (Adams et al. 2011). In addition to RTV, omission errors on a computerized attention task are common measures of distractibility (Adams et al. 2011; Bron et al. 2014; Tucha et al. 2006). Omission errors were found to be associated with ADHD symptomatology (Losier et al. 1996) and a recent study suggested links between movement disturbances attributable to dysfunction of the cerebellum and omission errors in childhood ADHD (Goetz et al. 2017). Thus, processing speed assessed by reaction times and distractibility assessed by the combination of RTV and omission errors can be proposed to reflect basic cognitive processes in adult ADHD and may interact with other functions to influence behavior.

Previous research highlighted the need to investigate how basic neuropsychological deficits converge to cause downstream effects on behavior (Adams et al. 2011; Mueller et al. 2017), for example, how increased RTV can manifest in risky driving. Adams et al. (2011) suggest that basic processes in response inhibition may interact with other neuropsychological functions, resulting in downstream effects in behavior. However, while neuropsychological deficits in patients with ADHD have already been identified at the basal level, the relationship between basic processes (e.g. distractibility and processing speed) and more complex cognitive functions (e.g., memory, executive functions) has not yet been investigated. If functions such as RT and RTV are among the basic processes underlying the cognitive functioning of adults with ADHD, the question arises in which way impairments of basic processes will account for higher order impairments, for instance in complex attention, executive function, and memory. Specifically, if an impairment in a lower order process such as RTV exists, any function which is building upon that and on a higher level of complexity may be affected (Felmingham et al. 2004; Ponsford and Kinsella 1992; Veltman et al. 1996). In ADHD, these basic processes may relate to core features of the disorder.

Furthermore, these basic processes may not only play a role in cognitive functions, but also in clinical outcomes. Stimulant drug treatment such as methylphenidate (MPH) is the first-line pharmacological treatment in adult ADHD and may reduce ADHD symptomatology and increase patients’ quality of life (Wigal 2009; Wigal et al. 1999; Wilens et al. 2001). In previous studies, patients with ADHD had faster RTs when treated with stimulants than when treated with placebos (Wong and Stevens 2012) and MPH also resulted in large decreases of RTV (Bron et al. 2014; Kofler et al. 2013). The finding that stimulant drug treatment in patients with ADHD affects RT and RTV further supports the notion that processing speed and distractibility may represent basic processes in adult ADHD. Moreover, an improvement of basic processes by MPH may result in meaningful changes in patients’ neuropsychological functioning.

The central focus of this study was to explore whether deficits in basic processes (distractibility and processing speed) may affect higher order functions (executive functions, complex attention, and memory) of adults with ADHD. To do so, several expectations were formulated: In line with previous findings, patients with ADHD without MPH were (1) expected to show impairments in higher order functions, namely complex attention, executive functioning, and memory. Furthermore, this study aimed to explore whether (2) a significant proportion of the impairment in these higher order functions can be explained by impairment of basic processes such as processing speed and distractibility. Moreover, we expect (3) an improvement in some cognitive domains in patients with ADHD treated with MPH compared to patients not treated with MPH. Improvements are expected in some but not all functions, as previous research found that although MPH improves a variety of functions, it does not normalize functioning (Fuermaier et al. 2017; Tucha et al. 2006; Turner et al. 2005). Finally, (4) if higher order functioning is improved as a result of treatment with MPH, it was expected that this improvement could be partially attributed to improvements in basic cognitive functions.

## Method

### Participants

*Patients with ADHD* Eighty-six patients with ADHD (55 without stimulant medication and 31 with stimulant medication) participated in this study. Recruitment occurred through self-referral or referral by local psychiatrists or neurologists to the Department of Psychiatry and Psychotherapy, SRH Hospital, Karlsbad-Langensteinbach, Germany. Qualified clinicians from the department performed the diagnostic assessment, which included a clinical psychiatric interview focusing on childhood and current symptomatology. The interview was conducted according to the Diagnostic and Statistical Manual of Mental Disorders criteria (DSM-IV; American Psychiatric Association 1994), as outlined by Barkley and Murphy (1998). All diagnoses were made by mutual agreement between at least two clinicians who were part of a diagnostic team and experienced in the assessment and treatment of adults with ADHD. The diagnostic assessment also included identifying objective impairments supporting the diagnosis of ADHD (e.g. evidence derived from school reports, failure in academic and/or occupational achievement) and comprised, if possible, multiple informants, such as employer evaluation and partner- or parent-reports. To investigate symptom severity, all participants filled out two standardized self-report scales assessing current and retrospective ADHD symptoms (Rösler et al. 2008). The short version of the Wender Utah Rating Scale (WURS-K, 25 items on a five-point scale) was used to assess self-reported childhood ADHD symptoms (Ward et al. 1993). For ADHD symptoms in adulthood, the ADHD Self-Report scale (ASRS; Adler et al. 2006, 2008; Kessler et al. 2005) was employed, which includes 18 items on a four-point scale and corresponds to DSM-IV diagnostic criteria (American Psychiatric Association 1994; Rösler et al. 2008). Patients treated with stimulant medication were asked to rate the severity of their symptoms when not under the influence of the medications. This allowed a comparison of self-reported symptom severity of both groups of patients disregarding the effect of medication.

The selection of patients was based on their diagnosis, age, intellectual functions (IQ), as well as their willingness to participate in the study. The 31 patients treated with stimulant medication were assigned to the ADHD-ON group, while the remaining 55 patients formed the ADHD-OFF group. For all patients, the exclusion criteria were as follows: (a) chronic medical conditions, (b) current or past psychosis, (c) substance abuse in the past 6 months, (d) history of neurological disorders including head injury and (e) verbal IQ estimate lower than 85. Due to these criteria, two patients of the ADHD-OFF group were excluded, as one had a neurological disorder and the other psychosis. Diagnostic subtypes of ADHD and comorbid psychiatric disorders are displayed in Table 1. The patients in the ADHD-ON group were treated with methylphenidate (MPH) on a daily basis and the mean dosage was 35.5 mg/day (dosages ranging from 10 to 80 mg daily). All treatment regimens with MPH were individually tailored and clinically appropriate.

**Table 1:** Characteristics of participants (mean ± standard deviation)

	Healthy participants (*n* = 80)	ADHD-OFF (*n* = 53)	ADHD-ON (*n* = 31)
Age (years)	34.2 ± 11.4	35.0 ± 10.7	33.9 ± 9.6
Gender (male/female)	41/39	27/26	19/12
Intellectual Functions (IQ)^a^	103.9 ± 11.7	101.3 ± 11.5	105.9 ± 12.3
ASRS	8.3 ± 5.7*	34.0 ± 8.2*	28.9 ± 11.2*
WURS-K	11.5 ± 8.7*	46.0 ± 12.8*	45.8 ± 11.7*
ADHD subtype^b^	–	64/28/2/6	71/23/3/3
Comorbidities^c^	–	28/8/4/2/2/0/0/0/0	32/6/6/3/0/3/3/6/3

*ADHD-OFF* patients with ADHD not treated with methylphenidate, *ADHD-ON* patients with ADHD treated with methylphenidate, *ASRS* ADHD self-rating scale, *WURS-K* Wender-Utah rating scale, short form

*Group comparisons comparing patients with controls, statistically significant at *p *< .001

^a^Multiple Choice Vocabulary Test (MWT-B)

^b^In %, i.e. combined/inattentive/hyperactive/unknown

^c^In %, i.e. mood disorders/personality disorders/anxiety disorders/addiction disorders/eating disorders/post-traumatic stress disorders/obsessive compulsive disorders/somatoform disorders/narcolepsy

*Controls* To form the control group (CG), a total of 80 individuals were included in the study. Recruitment occurred through public announcements, word of mouth and contacts of the researchers. At the clinical assessment, none of controls indicated any history of neurological or psychiatric disease or usage of medications affecting the central nervous system. To control for ADHD symptomatology, all control participants completed the same self-report scales for current and retrospective symptoms as patients. In addition, verbal IQ was estimated through a Multiple Choice Vocabulary Test (Lehrl 1995). Descriptives of controls as well as the two patient groups are displayed in Table 1. Group comparisons indicated no significant difference in age, *F* (2) = .131; *p *= .877, gender, *χ*^2^ (2)= 1.044; *p *= .593 and intellectual functions, *F* (2) = 1.63; *p *= .199. Patients scored significantly higher than controls on current symptoms [ADHD–SR, *F* (2) = 194.01; *p *< .001] and retrospective symptoms [WURS-K, *F* (2) = 209.01; *p *< .001], which corroborated their diagnostic status.

### Materials

#### Intellectual functions (vocabulary skills)

Intellectual functions were assessed with the Multiple Choice Vocabulary Test (MWT-B; Lehrl 1995). In this short, 37-item test for vocabulary skills participants had to select a real word that was intermixed with four made-up words. For each correctly selected item, the participant received a point and these points were summed up to the total score, which was compared to a normative sample and thereby transformed to an IQ score.

#### Attention

Attention functions were assessed with subtests of the test battery for Attention Performance (TAP, Zimmermann and Fimm 2008), i.e. the Alertness, Vigilance, Selective Attention, and Divided Attention subtests.

Tonic as well as phasic alertness (Zimmermann and Fimm 2008) was measured by presenting visual stimuli that were either preceded by an auditory cue (phasic) or displayed without prior warning (tonic). Participants were asked to respond as fast as possible to the target stimulus by pressing a specific button. The mean reaction time (RT) and the dispersion of the reaction time (SD) were recorded.

The vigilance test (Zimmermann and Fimm 2008) required participants to remain attentive over a prolonged period of time (30 min). A sequence of tones was administered, consisting of alternating high- and low-pitched tones. A target stimulus was presented at a rate of about 1 per 50 s. The target stimulus was defined as the interruption of this sequence by two tones of the same kind, either two high or low pitched tones, upon which the participant had to react as fast as possible by pressing a specific button. The mean reaction time (mean RT) and dispersion of reaction time (SD) were recorded for further analysis.

Selective attention was measured through a visual scanning task (Zimmermann and Fimm 2008) Participants were presented with a matrix of rectangles which opened in different directions. They were asked to scan the rectangles for a certain target rectangle and had to respond by pressing a specific button if the target rectangle was included or press another button if the target rectangle was not included. Number of omission errors and commission errors were recorded.

The divided attention test combined auditory and visual elements that participants had to attend to simultaneously (Zimmermann and Fimm 2008). For the visual part, between six and eight crosses were presented simultaneously that formed a pattern on the screen. If the crosses occurred at neighboring positions and thus comprised a square, the participant had to react as fast as possible by pressing a button. At the same time as the changing of the visual stimuli, a sequence of alternating high- and low-pitched tones was administered which constituted the auditory component. If a tone of the same type occurred twice, the participant had to react as fast as possible by pressing a button. The combination of the visual and auditory parts of this task was used to assess divided attention. Number of omission errors and commission errors were recorded for further analysis.

#### Executive functions

Interference was measured with the Stroop Color–Word test (Bäumler 1985; Stroop 1935). This test consisted of three parts: in the Color–Word condition, the words for colors (BLUE, GREEN, YELLOW, RED) printed in black were shown to participants who had to read them out as fast as possible. For the Color–Block condition, differently colored rectangles (in blue, green yellow and red) were presented and the participant had to name the color of the rectangle as fast as possible. Last, in the Color–Word interference condition, the words for colors were presented in a dissimilar color (e.g., the word GREEN printed in red ink) and participants had to name the color of the ink as fast as possible while ignoring the meaning of the word. The main variable of interest was an interference quotient calculated by dividing the response time (RT) of the Color–Word interference condition by the RT of the Color–Word condition.

Planning abilities were assessed with a task of delayed task execution (Fuermaier et al. 2013, 2017). This test consisted of ten separate subtests involving word finding, screwing, ball squeezing, arithmetic and cancellation tasks. Participants were asked to plan a sequence for executing the tasks and after a delay of 60 min were asked to carry out the previously formulated plan. While doing so, participants had to follow a number of rules: (a) observe a time limit of 10 min, (b) work on each subtest at least once, (c) adhere to a restricted sequence of tests, and (d) optionally work on two tasks simultaneously. This task allows the assessment of several cognitive components, namely planning, recall, self-initiation, and execution (as measured by switching, described under the memory subsection). To yield a score for planning, a scoring scheme was applied that considered various aspects of plan quality, such as adherence to rules, specifying a time sequence, simultaneous performance of two tasks and so on. A sum score was calculated to assess plan quality, with larger scores indicating better planning skills (for more details of the task, see Fuermaier et al. 2013).

For cognitive flexibility, the Trail Making Test (TMT; Reitan 1958) was used. In the first part of this test (trail A), participants were requested to connect numbers in ascending order by drawing lines as quickly as possible. For the second part (trail B), participants were again asked to draw lines as fast as possible, but this time they also had to switch between connecting numbers in ascending order and connecting letters of the alphabet in ascending order (e.g. 1-A-2-B-3-C). The RTs for both parts were registered. In order to get a measure of flexibility without the temporal component, a quotient was computed by dividing the RT for Trail B by the RT of trail A.

Verbal fluency was assessed with the Regensburger Word Fluency Test (RWT; Aschenbrenner et al. 2000), which required participants to produce as many words as possible during a two-minute time interval. These words had to begin either with the letter “H” or “T”, but each word that was named needed to follow an alternating H/T sequence. For example, “house, tulip, heart, tower” and so forth are correct, but for “hurricane, taste, tantrum, honey”, “tantrum” would have been counted as an error. In addition, words were counted as errors if they were names (e.g. Harry, Helsinki, etc.), had the same stem (e.g. test, test results, test anxiety), or were perseverations of words already mentioned (Aschenbrenner et al. 2000). The variable of interest for verbal fluency was the number of correctly produced words.

Working memory was measured with the Digit Span Backwards test, which is a subtest of the Wechsler Memory Scale (Wechsler 1987). Number sequences were read to the participant who was then requested to repeat the numbers in reverse. The main variable of interest was the number of correctly repeated sequences.

#### Memory

Immediate recall as well as delayed recall (episodic retrospective memory) was measured with the Logical Memory subtest of the Wechsler Memory scale (Wechsler 1987). In this test, two short stories were read to participants, who had to retell the stories immediately and after a 20-minute delay. For both immediate and delayed recall, the amount of correctly recalled elements of the stories was registered.

Immediate recognition (encoding) and delayed recognition (retention) were assessed with the Immediate and Delayed Recognition Tests (Fuermaier et al. 2013, 2017). Both tests were word list paradigms and included a study phase and a recognition phase. In the study phase, 40 unrelated nouns were presented for 4 s each on the center of a screen. Participants were asked to use whichever mnemonics they considered effective to remember the words. For the immediate recognition test, the recognition phase followed directly after the study phase. Participants were shown all words from the study list as well as 40 additional words and were asked to press one of two predefined buttons to indicate whether each word had been displayed or not previously. The delayed recognition test followed the same proceedings as the immediate recognition test with the exception that the recognition phase followed after a delay of 40 min. The main variable of interest was the number of correctly identified words for both tests.

Visuospatial memory was assessed with the Rey–Osterrieth Complex Figure (Osterrieth 1944; Rey 1941). Participants were given a complex geometrical figure and were asked to copy it as accurately as possible. The figure was taken away and after a delay of 25 min\; the participants were requested to draw the figure from memory. A scoring scheme was applied which gives two points for the correct reproduction of any of the 18 units per drawing: one point if the unit was drawn either incompletely or misplaced and half a point if the unit is distorted in both regards. The sum score for the delayed copy was registered as a measure of visuospatial memory.

For source memory, a source discrimination paradigm was employed (Fuermaier et al. 2013, 2017). This included the consecutive presentation of 28 unrelated German nouns (7 s each), half of which were presented in green letters on the right-hand side of the screen and the other half in blue on the left-hand side. The participants were asked to memorize the words as well as their location and corresponding color and were encouraged to use any mnemonics they could think of. Directly after the study phase, participants were shown the 28 words consecutively in black font in the center of the screen and had to indicate where and how the words were presented previously. The main variable of interest was the number of correctly classified words.

Prospective memory was assessed with a planning task requiring delayed task execution (Fuermaier et al. 2013). In this task, several components of complex prospective memory could be distinguished, i.e., planning, recall, self-initiation, and execution (for details of the task performed, see Fuermaier et al. 2013). However, general task performance was captured by a measure of switching between subtests (task switching). Task switching was calculated by summing up the number of subtests the participant actually initiated under consideration of rules. Task switching may indicate general task performance because in order to successfully execute the overall task, participants were required to actively switch between subtests. Therefore, the number of initiated subtests during task execution was used as the primary measure for prospective memory.

#### Distinction of basic and higher order cognitive processes

To explore the role of impairment in basic processes to impairment in higher order functions, the variables of the aforementioned tests were grouped into sets of predictors, as displayed in Table 2. Five sets of predictors were distinguished, namely processing speed and distractibility as sets of basic processes and executive functions, memory, and complex attention as sets of higher order functions. Variables were grouped based on the task demands and complexity of the neuropsychological tests. Processing speed and distractibility included variables of reaction time, variability of reaction time, and omission errors of basic cognitive tests, i.e. Alertness and Vigilance. These tests are considered as basic as they require participants to attend to one source of information only and to react to stimuli without considering external distracting or conflicting information. In addition, all measures grouped as basic processes can be classified under the ‘intensity’ category of attention and not the ‘selectivity’ category, which includes more complex elements (Kahneman 1973; Posner and Rafal 1987; van Zomeren and Brouwer 1994). Complex attention, executive functions, and memory included variables of higher order tasks that require participants to integrate the information provided (Table 2). Regarding the measures of higher order cognitive functions, reaction times and variability of reaction times were not considered as test variables.

**Table 2:** Neuropsychological test performance of patients with ADHD and the control group

Variables	CG	ADHD-OFF	ADHD-ON	Omnibus test (Kruskal–Wallis)		Pairwise group comparisons		
	(*n* = 80)	(*n* = 53)	(*n* = 31)			CG vs. ADHD-OFF	CG vs. ADHD-ON	ADHD-OFF vs. ADHD-ON
	M ± SD	M ± SD	M ± SD	*χ*^2^ (*df*)	*p*	Cohen’s *r*	Cohen’s *r*	Cohen’s *r*
Basic cognitive functions								
*Processing speed (Z score)* ^*a*^		− .*77*	− .*23*					
Tonic alertness (RT)	245 ± 44	271 ± 59	249 ± 38	8.76 (2)	.013	–	–	–
Phasic alertness (RT)	243 ± 45	260 ± 56	243 ± 32	4.69 (2)	.096	–	–	–
Vigilance (RT)	566 ± 117	722 ± 189	639 ± 154	25.93 (2)	< .001*	.44*	.21	.20
*Distractibility (Z score)* ^a^		− .*80*	− .*22*					
Tonic alertness (SD)	38 ± 24	55 ± 33	38 ± 20	13.61 (2)	.001*	.31*	.04	.27*
Phasic alertness (SD)	41 ± 22	48 ± 27	37 ± 15	4.10 (2)	.128	–	–	–
Vigilance (SD)	111 ± 51	176 ± 82	144 ± 99	23.67 (2)	< .001*	.42*	.15	.25
Vigilance (omissions)	1.01 ± 1.67	2.53 ± 3.70	1.83 ± 3.09	8.26 (2)	.016	–	–	–
Higher order cognitive functions								
*Executive functions (Z score)* ^a^		− .*55*	− .*20*					
Interference (quotient)	2.62 ± .50	2.79 ± .63	2.56 ± .54	3.69 (2)	.158	–	–	–
Planning (score)	20.78 ± 6.16	11.68 ± 5.86	15.36 ± 7.01	49.38 (2)	< .001*	.60*	.33*	.24
Cognitive flexibility (quotient)	2.48 ± .75	2.64 ± .81	2.37 ± .67	2.53 (2)	.282	–	–	–
Verbal fluency (# correct)	23.16 ± 6.29	20.79 ± 6.08	23.68 ± 6.35	4.76 (2)	.093	–	–	–
Working memory (# correct)	7.24 ± 2.11	6.49 ± 1.84	6.26 ± 1.57	7.38 (2)	.025	–	–	–
*Memory (Z score)* ^a^		− .*80*	− .*51*					
Immediate recall (# correct)	32.55 ± 6.13	23.68 ± 6.48	23.97 ± 6.16	55.37 (2)	< .001*	.58*	.51*	.09
Delayed recall (# correct)	29.01 ± 7.30	20.21 ± 7.02	20.32 ± 6.84	45.39 (2)	< .001*	.52*	.46*	.09
Immediate recognition (% correct)	83.37 ± 10.26	76.53 ± 12.43	81.24 ± 9.84	9.21 (2)	.010	–	–	–
Delayed recognition (% correct)	75.04 ± 12.28	67.31 ± 9.71	72.68 ± 11.46	13.85 (2)	.001*	.32*	.08	.23
Visuospatial memory (# correct)	20.04 ± 5.83	20.20 ± 6.60	25.00 ± 5.46	17.03 (2)	< .001*	.03	.38*	.38*
Source memory (# correct)	79.34 ± 13.89	69.27 ± 15.55	73.63 ± 15.79	12.78 (2)	.002*	.31*	.16	.13
Prospective memory (score)	12.55 ± 6.39	6.36 ± 4.06	6.19 ± 3.51	50.81 (2)	< .001*	.54*	.51*	.10
*Complex attention (Z score)* ^a^		− .*69*	− .*02*					
Selective attention (omissions)	8.71 ± 7.44	11.10 ± 8.61	9.00 ± 9.82	5.22 (2)	.074	–	–	–
Selective attention (commissions)	.31 ± .63	1.15 ± 5.73	.33 ± .76	.05 (2)	.975	–	–	–
Divided attention (omissions)	1.15 ± 1.30	2.44 ± 2.62	.80 ± 1.06	15.01 (2)	.001*	.27*	.11	.39*
Divided attention (commissions)	1.00 ± 1.26	1.12 ± 1.18	1.41 ± 1.61	1.06 (2)	.589	–	–	–

*CG* control group, *ADHD-OFF* patients with ADHD without methylphenidate, *ADHD-ON* patients with ADHD with methylphenidate treatment

Definition of sets of test measures assessing basic processes and higher order functions

Basic processes:

*Processing speed* Tonic alertness (RT), phasic alertness (RT), and vigilance (RT) = mean reaction time in ms of the respective attention subtest (TAP)

*Distractibility* Tonic alertness (SD), phasic alertness (SD), and vigilance (SD) = dispersion of reaction time in ms of the respective attention subtest (TAP). Vigilance (omissions) = number of omission errors of the vigilance test (TAP)

Higher order functions:

*Executive functions* Interference (quotient) = division response time Color–Word interference by response time Color–Word (Stroop Color–Word test). Planning (Score) = sum score (delayed task execution task). Cognitive Flexibility (Quotient) = division response time Trail B by response time Trail A (TMT). Verbal fluency (# correct) = number of correctly produced words (RWT). Working memory (# correct) = number of correctly repeated sequences (Digit Span Backwards test)

*Memory* Immediate recall (# correct) and Delayed recall (# correct) = number of correctly recalled story elements of the Logical Memory subtest (Wechsler Memory scale). Immediate recognition (% correct) and delayed recognition (% correct) = number of correctly identified words (Immediate and Delayed Recognition tests). Visuospatial memory (# correct) = sum score delayed copy (Rey-Osterrieth Complex Figure). Source memory (# correct) = number of correctly classified words (source discrimination paradigm). Prospective memory (Score) = number of initiated subtests (delayed task execution task)

*Complex attention* Selective attention (omissions) and selective attention (commissions) = number of omission and commission errors of the selective attention test (TAP). Divided attention (omissions) and divided attention (commissions) = number of omission and commission errors of the divided attention test (TAP)

*Statistically significant at *p *< .01. Pairwise comparisons were computed only for significant effects on the omnibus test: a small effect is ≥ .1, a medium effect is ≥ .3 and a large effect is ≥ .5

^a^Composite score calculated by averaging *Z* scores of test variables for each set of predictors (based on healthy controls). Negative *Z* scores indicate impairment as compared to the control group

### Procedure

The current study was part of a larger research project and parts of the data were already used to answer other research questions (Fuermaier et al. 2015, 2017).

Each participant was tested individually. Prior to the assessment, each participant gave written informed consent. Participation was voluntary and not rewarded. The participants filled out the self-report scales to assess symptomatology prior to the administration of the neuropsychological tests. The order of the immediate and delayed word recognition test was counterbalanced to control for learning effects. Interference was avoided by administering the vigilance test (a nonverbal test) in the delay between the study phase and the recognition phase of the delayed recognition test. In the second part of the assessment, participants completed the remaining cognitive tests, including the story recall (in order to separate verbal memory tests from each other). In the delay between the first part of the story recall test and the delayed story recall, participants performed nonverbal tests assessing executive functions and attention. Afterwards, participants were debriefed and thanked for their participation at the end of the study. In total, the duration of the assessment was about 2.5 h per participant.

### Ethics statement

The ethics committee of the medical faculty of the University of Heidelberg, Germany, approved the study and its adherence to ethical standards. This research project was conducted in accordance with the Helsinki Declaration.

### Statistical analysis

Firstly, test scores of cognitive functions were compared between the groups. To yield a composite *z*-score for each set of predictors (see materials section “Distinction of Basic and Higher Order Cognitive Processes”), the *z*-scores for each variable of each set were calculated. Variables for which a positive *z*-score indicated impairment were recoded to ensure that for all variables, a negative *z*-score indicated impairment as compared to the control group. Next, the average of all z-transformed variables per set of predictors was calculated to represent the composite score per domain. Spearman’s correlation coefficients (non-parametric alternative to Pearson’s product-moment correlation coefficients) of the composite scores and ASRS scores were computed to explore the association with ADHD symptom severity. Furthermore, boxplots were created to display the heterogeneity of cognitive performance within the two patient groups.

As normality and homoscedasticity assumptions were violated in several instances, the Kruskal–Wallis test was chosen as a non-parametric alternative to the Analysis of Variance tests. The significance level was adjusted to *p *< .01 in order to control for alpha error inflation due to multiple testing. Dunn’s tests with an alpha correction for multiple comparisons were employed as a follow-up pairwise analysis. This allowed the direct comparison of groups on individual test measures of cognitive performance. Furthermore, effect sizes (Cohen’s *r*) of all significant group differences were computed. Cohen’s *r* was chosen as it does not rely on the normality assumption. Based on Cohen’s criteria for *r*, .1 indicates a small effect, .3 indicates a medium effect, and .5 indicates a large effect (Cohen 1988). Binomial logistic regression analyses were conducted to determine the size of cognitive impairment of patients with ADHD compared to controls. Logistic regression analyses were carried out separately for the comparison of the ADHD-OFF group with the CG and the ADHD-ON group with the CG as the dependent variable. To investigate the contribution of impairment in basic processes to impairment in higher order functions in the two patient groups, individual test measures were grouped into sets of predictor variables (see “Materials” section “Distinction of basic and higher order cognitive processes”), representing processing speed, distractibility, executive functions, memory, and complex attention (see Table 2 for a complete presentation of predictor variables). Sets of basic processes (processing speed or distractibility) and sets of higher order functions (executive functions, memory or complex attention) were entered as predictors. First, multiple regression analyses were conducted in order to determine the level of impairment of patients with ADHD as compared to controls in higher order cognitive processes, i.e. executive functions, memory, and complex attention. Next, to investigate the effect of impairments in basic processes on impairments in higher order processes, hierarchical regression models were computed that included basic cognitive functions (processing speed or distractibility) in step 1 and complex cognitive functions (executive functions, memory or complex attention) in step 2, with patient status (CG vs ADHD-OFF or ADHD-ON) as the outcome variable. Pseudo *R*^2^ values were calculated for each logistic regression model and were interpreted as effect size estimators of the impairment of patients with ADHD in each set of functions. Nagelkerke’s *R*^2^ was chosen as it adjusts for the differential scaling of the commonly used Cox’s and Snell *R*^2^ and is thus more readily interpretable. Furthermore, a Bonferroni adjusted significance level of *p *< .005 was applied in the regression analyses to control for problems in multiple testing.

## Results

### Neuropsychological functioning in patients with ADHD and controls

**Fig. 1:**
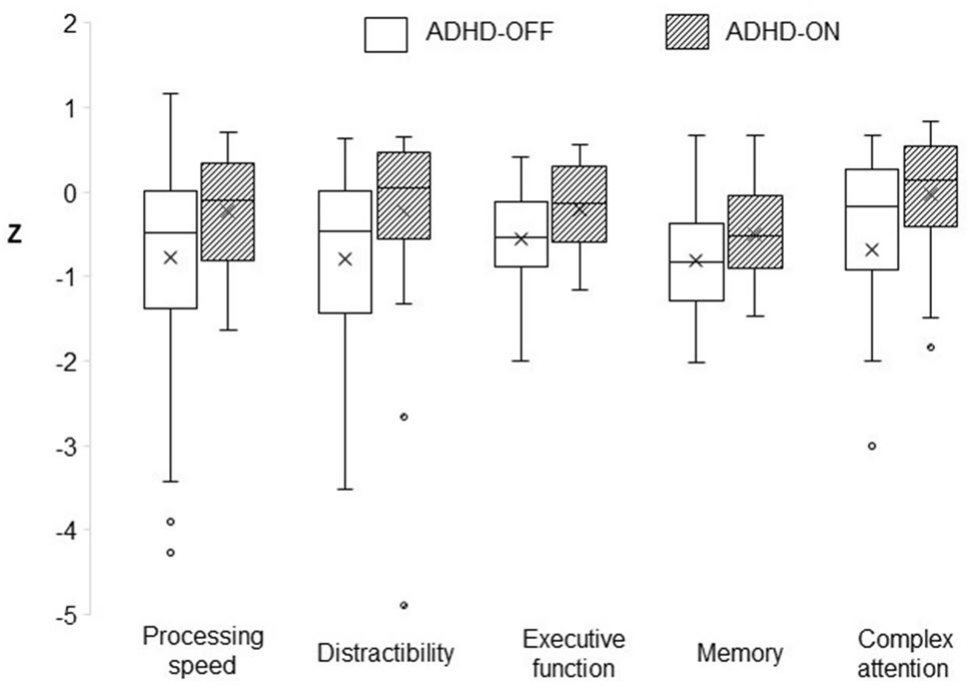
Variability of composite scores of cognitive test variables of the ADHD-OFF and ADHD-ON groups (compared to CG). *ADHD-OFF* patients with ADHD without methylphenidate treatment, *ADHD-ON* patients with ADHD with methylphenidate treatment, *CG* control group. Circles denote outliers. One outlier in complex attention of the ADHD-OFF group was removed (*Z* = − 17). The cross indicates the mean

The composite *z*-scores demonstrated impairments in patients with ADHD without MPH and less pronounced deficits in patients with MPH in both basic cognitive functions and higher order cognitive functions (Table 2). The heterogeneity of these cognitive impairments is displayed in Fig. 1. Spearman’s correlations between composite scores and the ASRS are presented in Table 3.

**Table 3:** Spearman correlation coefficients between composite scores of cognitive test variables and the ADHD Self-Report Scale (ASRS)

Group	Processing speed	Distractibility	Executive functions	Memory	Complex attention
CG	− .123	− .106	− .154	− .025	.072
ADHD-OFF	− .028	− .012	− .149	.093	− .126
ADHD-ON	− .244	.202	.213	.088	.140

None of the correlations reached statistical significance (all *p* values > .05)

*ADHD-OFF* patients with ADHD not treated with methylphenidate, *ADHD-ON* patients with ADHD treated with methylphenidate, *CG* control group

Kruskal–Wallis tests revealed significant differences between patients with ADHD and control participants on individual test measures (Table 2). The pairwise comparisons (Dunn’s tests) of the ADHD groups to controls and corresponding effect sizes indicated impairments on individual test measures ranging from small to large effect sizes. In comparison to the CG, the ADHD-OFF group showed significantly poorer performances with regard to processing speed (medium effect for vigilance RT) and distractibility (medium effects for tonic alertness SD and vigilance SD) as well as executive functions (large effect for planning), memory (medium to large effects for immediate recall, delayed recall, and prospective memory, delayed recognition, and source memory) and complex attention (small effect for divided attention omissions). Compared to the CG, the ADHD-ON group showed no significant impairment in processing speed, distractibility, and complex attention. Significantly poorer performance of the ADHD-ON group compared to CG was found for executive functions (medium effect for planning) and memory (medium to large effects for immediate recall, prospective memory, delayed recall and visuospatial memory).

### Contribution of basic processes to impairment in higher order functions

To investigate the contribution of impairment in basic processes to impairment in higher order functions in the two patient groups, five sets of predictors were distinguished, namely processing speed and distractibility as sets of basic processes and executive functions, memory, and complex attention as sets of higher order functions. Hierarchical regression models were computed in which step 1 included basic cognitive functions (processing speed or distractibility) and in step 2 complex cognitive functions (executive functions, memory or complex attention), with patient status (CG vs ADHD-OFF or ADHD-ON) as the outcome variable (Tables 4, 5, respectively). For each set of higher order functions, a regression analysis revealed a significant impairment in the ADHD-OFF group compared to CG, i.e. in executive functions (*R*^2^ = .467), memory (*R*^2^ = .582), and complex attention (*R*^2^ = .150). In order to delineate to what extent impairments in basic processes account for impairments in higher order functions, hierarchical logistic regression models were employed. Whereas a model including processing speed revealed an impairment of *R*^2^ = .305, a hierarchical model including both processing speed and executive functions demonstrated an effect size of cognitive impairment of *R*^2^ = .579, indicating that executive functions increased the size of impairment by *R*^2^ = .274 in addition to impairments in processing speed. Compared to a model that only includes executive functions (*R*^2^ = .467), it can be concluded that processing speed accounted for about 41% (drop of *R*^2^ from .467 to .274) of the impairment in executive functions of patients with ADHD. Following this approach, impairments in distractibility (*R*^2^ = .318) accounted for *R*^2^ = .201 (drop of *R*^2^ from .467 to .266, so 43%) of the impairment in executive functions. With regard to memory, processing speed (*R*^2^ = .304) explained 29% (*R*^2^ = .582–.414) and distractibility (*R*^2^ = .308) explained 27% (*R*^2^ = .582–.425) of the impairments in memory functions of adults with ADHD. In complex attention, processing speed (*R*^2^ = .300) explained 56% (*R*^2^ = .150–.066) and distractibility (*R*^2^ = .297) explained 74% (*R*^2^ = .150–.039) of the impairment.

**Table 4:** Logistic regression analyses indicating cognitive impairment of patients with ADHD without stimulant treatment (ADHD-OFF) compared to healthy controls (CG)

Predictor sets	*χ*^2^ model	*df*	*p*	*R*^2^ total	*R*^2^ higher order functions^d^
Executive function					
Model 1^a^	55.856	5	< .001*	.467	
Model 2^b^	73.559	8	< .001*	.579	.274
Model 3^c^	74.026	9	< .001*	.584	.266
Memory					
Model 1^a^	74.418	7	< .001*	.582	
Model 2^b^	101.012	10	< .001*	.718	.414
Model 3^c^	102.497	11	< .001*	.733	.425
Complex attention					
Model 1^a^	14.965	4	.005*	.150	
Model 2^b^	40.149	7	< .001*	.366	.066
Model 3^c^	36.298	8	< .001*	.336	.039

The *R*^2^ represents a summary measure reflecting the overall impairment in the various cognitive variables (see materials section “Distinction of basic and higher order cognitive processes”)

*ADHD-OFF* patients with ADHD without methylphenidate treatment, *CG* control group

*Statistically significant at *p *< .005

^a^Basic model including only variables of higher order functions

^b^Model with variables of processing speed entered in step 1 and variables of higher order functions entered in step 2

^c^Model with variables of distractibility entered in step 1 and variables of higher order function entered in step 2

^d^Impairment in higher order functions after controlling for impairments in lower order functions

**Table 5:** Logistic regression analyses indicating cognitive impairment of patients with ADHD treated with MPH (ADHD-ON) compared to healthy controls (CG)

Predictor sets	*χ*^2^ Model	*df*	*p*	*R*^2^ total	*R*^2^ higher order functions^d^
Executive functions					
Model 1^a^	19.609	5	.001*	.233	
Model 2^b^	28.239	8	< .001*	.328	.237
Model 3^c^	28.015	9	.001*	.328	.239
Memory					
Model 1^a^	67.995	7	< .001*	.663	
Model 2^b^	79.355	10	< .001*	.748	.661
Model 3^c^	69.333	11	< .001*	.683	.595
Complex attention					
Model 1^a^	6.961	4	.138	.094	
Model 2^b^	9.824	7	.199	.131	.080
Model 3^c^	11.818	8	.159	.156	.077

The *R*^2^ represents a summary measure reflecting the overall impairment in the various cognitive variables (see “materials” section “Distinction of basic and higher order cognitive processes”)

*ADHD-ON* patients with ADHD with methylphenidate treatment, *CG* control group

*Statistically significant at *p *< .005

^a^Basic model including only variables of higher order functions

^b^Model with variables of processing speed entered in step 1 and variables of higher order functions entered in step 2

^c^Model with variables of distractibility entered in step 1 and variables of higher order function entered in step 2

^d^Impairment in higher order functions after controlling for impairments in lower order functions

Logistic regression models of cognitive impairment with group status (ADHD-ON versus CG) as dependent variable are presented in Table 5. Regression analyses including only the higher order function showed significant impairments in the ADHD-ON group in executive functions (*R*^2^ = .233) and memory (*R*^2^ = .663), but not in complex attention (*R*^2^ = .094; *p *= .138). Whereas impairment was substantially lower in the ADHD-ON group than the ADHD-OFF group in executive function (*R*^2^ = .233 vs. .467) and complex attention (*R*^2^ = .094 vs. .150), impairments in memory showed a mild increase (*R*^2^ = .663 vs. .582). Regarding the effect of impairment in basic cognitive functions on impairment in higher order functions, processing speed did not explain any impairment in executive functions. The level of impairment in executive functions changed only marginally (*R*^2^ = .233–.237) when processing speed was already included in the model. Similarly, distractibility did not explain any impairment in executive functions, as the impairment in executive functions changed only slightly with distractibility included in the model (*R*^2^ = .233–.239). Processing speed influenced the impairment in memory only marginally (*R*^2^ = .663–.661) and distractibility (*R*^2^ = .663–.595) explained 10% of the memory impairment of the ADHD-ON group. Finally, no significant models were obtained for complex attention, indicating that the ADHD-ON group did not show any significant impairments in complex attention when compared to the CG (Table 5).

## Discussion

The aim of this study was to investigate whether impairments in basic cognitive processes can explain impairments in higher order functions in adults with ADHD. The results confirmed this prediction as impairments in processing speed and distractibility (basic processes) accounted for a considerable part of the impairment of the higher order processes (executive functioning: 41% for processing speed and 43% for distractibility, memory: 29% and 27%, complex attention: 56% and 74%). Thus, the current evidence adds to previous literature (Adams et al. 2011; Mueller et al. 2017; Mulder et al. 2010) suggesting that deficits in processing speed and distractibility appear to be core features of ADHD and impairment in these basic processes can lead to deficits in more complex functions.

### Cognitive impairment in patients with ADHD without stimulant treatment

To explore this matter, the first step was to establish whether patients with ADHD not treated with MPH show impairments in basic processes. Indeed, patients with ADHD showed impairments in processing speed (*Z* = − .77) and distractibility (*Z* = − .80; as measured by reaction time variability and omission errors). This is in line with a large body of research indicating slower reaction times and greater reaction time variability in adults with ADHD (Adams et al. 2011; Cross-Villasana et al. 2015; Gmehlin et al. 2016; Kofler et al. 2013; Leth-Steensen et al. 2000; Mostert et al. 2015). The next step was to establish the level of impairment in higher order functions. Significant deficits were found in all higher order functions, namely in executive functioning (*Z *= − .55; *R*^2^ = .467 for all tests of executive functioning included in the logistic regression model), memory (*Z *= − .80; *R*^2^ = .582), and complex attention (*Z* = − .69; *R*^2^ = .150), as compared to controls. With regard to individual tests, large differences were found between patients with ADHD and controls concerning planning, immediate and delayed recall as well as prospective memory (see Table 2). The size of these impairments was mostly in line with previous research, with the apparent exception of the observed impairment in complex attention, which was lower than otherwise reported (Schoechlin and Engel 2005). One could consider this a contradiction, but upon closer look it becomes clear that the differences may arise due to the selection of tests. In the present study, the Trail Making Test, Digit Span Backwards, and Stroop tests were considered tests of executive functioning, whereas Schoechlin and Engel (2005) grouped them under complex attention. If this is taken into consideration, the results of the current study align with Schoechlin and Engel (2005) in that a larger effect size for executive functioning and a smaller effect size for complex attention were found than in the Schoechlin and Engel (2005) study.

Given the current findings, the role of memory impairments in adults with ADHD should be emphasized, as impairments were found in multiple aspects of memory and across a variety of tests. These memory impairments were all medium to large in size. It is worth noting that prospective memory entails an aspect of executive functioning, so the grouping amongst memory function might be questioned. Nevertheless, the “classic” memory functions such as immediate and delayed recall also showed large effects, so the overall level of memory impairment would have been large even if prospective memory was not included. Although substantial memory deficits in adults with ADHD have been found in previous research (Fuermaier et al. 2017; Johnson et al. 2001; Lundervold et al. 2019; Rhodes et al. 2012; Schoechlin and Engel 2005), clinicians appear not to consider memory impairments particularly relevant in adult ADHD, as reported in a recent consensus report among clinicians and researchers working with adults with ADHD (Fuermaier et al. 2019). Interestingly, Lundervold et al. (2019) explored whether verbal memory deficits in adults with ADHD can be explained by impairments in working memory and response inhibition. Response inhibition in particular contributed to various aspects of memory and the authors emphasized the need for research investigating the overlap between cognitive functions.

### Impact of basic processes on higher order functions

After establishing the level of impairment in both basic processes and higher order functions, the next step underpinned the central question of this study, namely whether a part of the impairment in these higher order functions can be attributed to impairment in the basic processes. Indeed, we found that impairment in basic processes explained a considerable proportion of impairment in the higher order functions (41–43% in executive functions, 27–29% in memory and 56–74% in complex attention). Although no previous studies have explicitly investigated the link between impairments in basic processes and higher order functions in adults which ADHD, a few studies have investigated related elements. Fair et al. (2012) conducted a multivariate pattern analysis to shed light on the heterogeneity of cognitive impairment in ADHD by identifying neuropsychological subgroups in children with ADHD and typically developing children. Their factor analysis found RTV to be atypical in the ADHD group, which supports the notion that response variability is at the core of neuropsychological impairment in ADHD. Sonuga-Barke et al. (2010) identified distinct components of neuropsychological impairment (temporal processing, inhibitory control and delay related deficits) in children with ADHD. As these authors included variables of RT and RTV in the measures of temporal processing and delay-related deficits, the question arises whether the basic processes of the present study would be closely linked to the measures of Sonuga-Barke et al. (2010), when all measures would be administered in a comprehensive assessment. In general, it would be interesting to investigate the cognitive heterogeneity of ADHD (Mostert et al. 2015) under consideration of the effect of basic processes. For example, as basic processes seem to be at the core of cognitive impairment in ADHD, one could argue that basic processes may be equally affected in the different symptom presentations of ADHD. Alternatively, patients with the inattentive symptom presentation may show greater impairments in processing speed and patients with the hyperactive/impulsive symptom presentation in distractibility (Tucha et al. 2008). To explore the relationship between executive functions and other cognitive abilities, Salthouse (2005) conducted a factor analysis on a set of cognitive functions in a large sample of controls (*N* = 6959). The author found that executive functioning as well as other neuropsychological measures (e.g. vocabulary ability) were closely related to reasoning but also perceptual speed. It is especially noteworthy that this relationship between perceptual speed and higher order processes was found in a sample of healthy participants and not in a clinical population. It follows from the evidence enumerated above, that processing speed represents a basic process that may underlie a variety of higher order functions, and maybe this holds true not just for adults with ADHD but represents a more general cognitive mechanism that is also applicable to other populations.

Recently, evidence for the pervasiveness of impairments in RTV and processing speed in ADHD has been growing (Cross-Villasana et al. 2015; Gmehlin et al. 2014, 2016; Kofler et al. 2013) and relations between these basic processes and other functions are receiving research attention. Weigard and Huang-Pollock (2017) found that processing speed predicts working memory performance in children with ADHD. Mulder et al. (2010) identified a speed–accuracy tradeoff as a basic process in children with ADHD, although it is still to be investigated whether these findings also hold true for adults with ADHD. Nevertheless, the current study extends their line of arguments by not just identifying basic processes, but also investigating how impairments in these basic processes affect higher order functions. To visualize this, the analogy of a tower comes to mind: if the foundation (basic processes) is damaged; this will affect the stability of all structures that are built on top (higher order functions). However, not all parts may be equally affected (the leaning tower of Pisa is also still standing). In our study, impairments in basic processes most explained impairments in complex attention and least explained impairments in memory. It is worth noting that this may have been affected by the properties of the tests involved: For basic processes and complex attention, computerized tests were used, whereas for executive functions paper and pencil tests were employed and for memory a mix of both. This was due to the importance of precision in measuring reaction times and standard deviations for the attention tests. For the other tests, the clinical standard was paper and pencil and better normative data were available for those versions (Schmand 2019). Accordingly, computerized tests (which the basic processes are based on) may be better at predicting performance in other computerized tests (complex attention) than in paper and pencil tests (executive functioning). Nevertheless, the finding that basic processes most explained impairments in complex attention and least in memory also seems logical if one considers the neuropsychological components involved: cognitive functions required for complex attentional tasks may load heavily on processing speed due to the similarity of the task at hand, whereas in memory tasks a number of other processes may be required (e.g. verbal functions). To return to the tower analogy, complex attention may be seen as the blocks built up on the damaged part of the foundation (basic processes), whereas memory only partially builds on this damaged part but is also half built on another section of the foundation.

### MPH and cognitive functioning

The investigation of the effects of pharmacological treatment with MPH on cognitive functions provided converging evidence to the role of basic processes. Salum et al. (2014) noted an important limitation to understanding the role of processing speed for cognitive functioning in adults with ADHD. These authors pointed out that previous research mostly utilized a mixed group of medicated and non-medicated patients, which may have concealed the effects of processing speed on higher order functions. In the present study, patients treated with MPH showed no significant impairments in basic processes. This is in line with previous research indicating that MPH decreases reaction time variability and improves processing speed (Bron et al. 2014; Kofler et al. 2013; Wong and Stevens 2012) and supports our conceptualization of impairments in processing speed and distractibility as basic processes that may constitute at least part of the core of cognitive impairment in ADHD. Regarding higher order functions, the size of impairment in patients with ADHD treated with MPH was considerably smaller for executive functioning and complex attention compared to non-medicated patients with ADHD. However, the impairment in several aspects of memory was found to be similar in patients treated with MPH as compared to patients with ADHD not treated with MPH. In the individual tests (univariate), patients with ADHD treated with MPH were mostly not impaired as compared to controls (see Table 2). This stands in contrast to regression models that included several functions of the same cognitive domain (multivariate), which showed significant impairments in executive functioning and memory, but not in complex attention (Table 5). This implies that although patients treated with MPH improved in their performance on individual tests, lingering deficits can still be observed in the multivariate analyses.

These findings are in line with previous research indicating that MPH improves but does not normalize cognitive functioning in adults with ADHD (Fuermaier et al. 2017; Tucha et al. 2006, 2011), and some impairment in various cognitive domains may remain despite treatment. It was predicted that part of the improvement in higher order functions could be attributed to improvement in basic cognitive functions. Indeed, for patients treated with MPH, basic processes were improved to the extent that no impairment could be found as compared to controls. Accordingly, basic processes did not explain a significant contribution of the impairment in higher order functions anymore (with the exception of distractibility explaining 10% in memory). Therefore, as basic functions improved, and higher order functions improved, it can be argued that the impairment that was explained by the basic functions is now removed from the higher order function. In brief, the improvement in the higher order function may partially be attributed to improvements in basic processes. Drawing on the tower analogy again, if we repair the foundation, then also the structures building upon it will become more stable.

### Implications for clinical practice

In future research, it is worth exploring exactly how this foundation is composed, to identify which basic processes constitute this foundation, which of them are functioning well and which are impaired in adults with ADHD. This information could be used to inform treatment strategies, as specifically targeting basic processes may lead to improvements in other cognitive domains. Thus, the implications for clinical practice are twofold. Firstly, the information that basic cognitive functions may constitute the foundation of cognitive impairment would allow clinicians to optimize the clinical assessment of adults with ADHD. Currently, large test batteries are commonly being used and there is little consensus as to which tests need to be included (Fuermaier et al. 2019). Lengthy testing can be very straining for the client as it requires considerable time and mental effort. Moreover, the widespread use of a multitude of neuropsychological tests to assess the wide range of impairments in adults with ADHD (Fuermaier et al. 2019) may be suboptimal as impairment in higher order functions seems to partially depend on impairment in basic processes. There is large overlap between the functions assessed by many neuropsychological tests and many of these tests seem to load heavily on processing speed (Salthouse 2005). By prioritizing tests assessing basic cognitive processes, it may be possible to shorten the selection of tests that are administered in a clinical neuropsychological assessment on patients with ADHD. Secondly, as basic processes seem to form the building blocks of higher functions building up on them, basic processes may serve as a target for treatment intervention. As the impairment in the basic process is reduced, any function that is building on this basic process may also show improvements. Cognitive training was discussed with regard to its’ potential to improve patients’ cognitive functioning (Sonuga-Barke et al. 2013) and directly targeting processing speed and distractibility may positively affect the patients’ cognitive functioning overall. Furthermore, the results of the current study showed that MPH administration may remove the impairment in basic processes. Thus, impairment in basic processes could serve as an indication for clinicians that MPH administration could be beneficial. It would also be worthwhile to explore how other common medication for ADHD, such as atomoxetine, affect basic processes. Of note, no significant correlations were found between ASRS scores and the composite scores of cognitive performance. This may reflect the discordance between subjective evaluation of cognitive symptoms by patients themselves (ASRS) and cognitive performance as measured by standardized testing (e.g. see Fuermaier et al. (2015) for a detailed account on the lack of overlap between cognitive performance evaluation gained from test measures and self-reports in adults with ADHD).

### Limitations and directions for future research

Several limitations of the present study must be noted. As this study did not employ a within subjects design on patients treated and not treated with MPH, the findings regarding the pharmacological treatment with MPH should be interpreted with caution. There may be qualitative differences between the two groups with regard to symptom presentations and comorbidities. This may partly be explained by the context that MPH is not randomly administered but depending on the clinical characteristics of the individual patient. Thus, no causal relationship regarding the effects of MPH on cognition can be established. It would be interesting for future research to replicate the study by employing a within subjects design and also to explore whether basic processes are differentially affected in the different symptom presentations of ADHD. For example, one may speculate that processing speed plays a larger role in the inattentive versus the hyperactive symptom presentation. Furthermore, the results of the study may be affected by the selection of the neuropsychological tests. In addition, considerable overlap may exist between tests (Salthouse 2005), which may be reflected in the similarity of the results for models (e.g. processing speed explaining 41% and distractibility explaining 43% of impairment in executive functions), so replicating this study with different neuropsychological tests assessing a similar set of functions as in the present study would add convergent validity. It has been proposed that RT and RTV may be susceptible to multicollinearity, implying that there may exist an overlap between the two basic processes identified in this study (Kofler et al. 2013). Although we based the allocation of basic cognitive processes into predictor sets on the most robust findings in the current literature (Kofler et al. 2013; Mueller et al. 2017), previous research indicated that slowing in RT could be explained by RTV (Kofler et al. 2013). For this reason, it is important to identify other basic cognitive processes. It would be interesting to delineate how such basic processes may interact to affect more complex, higher order cognitive functions. To investigate this matter, future research could conduct a factor analysis based on a large sample of patients with ADHD, which would add important information on how basic processes and the higher order functions relate to each other.

## Conclusion

In conclusion, the present findings highlight the importance of basic processes in the cognitive functioning of adults with ADHD. Basic processes seem to constitute at least part of the foundation upon which higher order functions are built upon. Consequently, impairments in these basic processes may have repercussions for a variety of higher order functions. This is also supported by the finding that a pharmacological treatment with MPH appears to improve basic processes and it could be argued that the improvements in basic processes partially account for improvements in higher order functions. Accordingly, utilizing this information in clinical settings could allow us to optimize the assessment of ADHD in adulthood. Basic processes may constitute a good target for treatment interventions, as improving basic processes may result in improvements in other cognitive functions as well. Designing and administering cognitive training interventions or medications that directly target processing speed and distractibility could generalize to improvements across a number of higher cognitive domains.
